# Genome Sequences of a Rat Polyomavirus Related to Murine Polyomavirus, *Rattus norvegicus* Polyomavirus 1

**DOI:** 10.1128/genomeA.00997-15

**Published:** 2015-09-03

**Authors:** Bernhard Ehlers, Dania Richter, Franz-Rainer Matuschka, Rainer G. Ulrich

**Affiliations:** a Division 12 “Measles, Mumps, Rubella and Viruses Affecting Immunocompromised Patients,” Robert Koch Institute, Berlin, Germany; b Environmental Systems Analysis, Institute of Geoecology, Technische Universität Braunschweig, Braunschweig, Germany; c Potsdam University Outpatient Clinic, Potsdam University, Potsdam, Germany; d Institute for Novel and Emerging Infectious Diseases, Friedrich-Loeffler-Institut, Greifswald-Insel Riems, Germany

## Abstract

We amplified and sequenced six complete genomes of a polyomavirus from feral Norway rats (*Rattus norvegicus*) and from a long-term breeding colony derived from Norway rats. This virus, which is closely related to hamster polyomavirus and murine polyomavirus, may contribute to understanding the evolutionary history of rodent polyomaviruses.

## GENOME ANNOUNCEMENT

Polyomaviruses (PyVs) infect various mammals, birds, and marine fish (1, 2). Four distinct PyVs have been identified in rodent hosts, murine PyV (MPyV), mouse pneumotropic virus (MPtV), hamster PyV (HaPyV), and *Mastomys* PyV (MasPyV) (3–6), whose full genomes are available in the GenBank database (http://www.ncbi.nlm.nih.gov/genbank/index.html). Here, we report the detection of a novel PyV in the spleen of 22/33 Norway rats (*Rattus norvegicus*) by generic PCR amplification of a VP1-encoding sequence of 215 bp according to a previously published protocol (7) and subsequent specific PCR. Twenty-four feral animals originated from a population in North Rhine-Westphalia (NRW), Germany, that has been previously tested to be free of rat hepatitis E virus infection (8). An additional nine animals were obtained from a breeding colony originally derived from animals captured at another location in NRW. This finding indicates that the virus naturally circulates in feral Norway rats and seems to be maintained in a feral rat-derived breeding colony. The short sequences were used to design back-to-back primers with which we amplified the rest of the six circular genomes (5,318 bp). The obtained PCR products were sequenced by a primer walking strategy. Among the six genomes, 18 single nucleotide polymorphisms (SNPs) were identified, and two genome pairs were completely identical. The virus was tentatively named *Rattus norvegicus* polyomavirus 1 (RnorPyV1). Previously, a PyV was detected in athymic rats (9), but a comparison to our novel sequences was not possible since sequences of this virus are not available. Interestingly, recent next-generation sequencing-based approaches in fecal samples from Norway rats failed to detect PyV sequences (10, 11).

The complete RnorPyV1 sequences display the typical genome organization of polyomaviruses with an early region encoding spliced small T (STAg) and large T (STAg) antigens and a late region encoding the structural VP1, VP2, and VP3 proteins, separated by a noncoding control region (NCCR). Like MPyV and HaPyV, RnorPyV1 also encodes a spliced middle T antigen (MTAg). The 18 SNPs are distributed over NCCR (*n* = 2), VP1 (*n* = 5), VP2/VP3 (*n* = 1), and the second exon of MTAg /LTAg coding sequences (*n* = 10), but not first exon of MTAg / LTAg or first and second exon of STAg. Five of the 18 SNPs resulted in 7 amino acid substitutions in the putative VP1 (*n* = 1), LTAg (*n* = 4), and MTAg (*n* = 2) proteins. A BLAST search revealed that RnorPyV1 is most similar to HaPyV and MPyV (respectively, 87% and 67% VP1 amino acid sequence identity). Phylogenetic analyses based on LTAg coding sequences indicated RnorPyV1 to be a sister taxon to a clade comprising MPyV and HaPyV, but not MasPyV and MPtV. We infer that the identification of such rodent viruses will help to elucidate the role of codivergence in the evolution of PyVs (12).

### Nucleotide sequence accession numbers. 

The complete genomes of RnorPyV1 have been deposited in GenBank under the accession numbers KR065723, KR065724, and KR075943 to KR075946.
